# Comparison of menaquinone-4 and menaquinone-7 bioavailability in healthy women

**DOI:** 10.1186/1475-2891-11-93

**Published:** 2012-11-12

**Authors:** Toshiro Sato, Leon J Schurgers, Kazuhiro Uenishi

**Affiliations:** 1 Fine Chemical Laboratory, J-OIL MILLS, INC, 1746 Nakashinden, Fukuroi-city, Shizuoka, 437-1111, Japan; 2 Department of Biochemistry, Cardiovascular Research Institute, University Maastricht, Maastricht, The Netherlands; 3 Laboratory of Physiological Nutrition, Kagawa Nutrition University, 3-9-2, Chiyoda, Sakado, Saitama, 350-0288, Japan

**Keywords:** Vitamin K_2_, Menaquinone-4, Menaquinone-7, Bioavailability, Absorption

## Abstract

**Background:**

Vitamin K_2_ contributes to bone and cardiovascular health. Therefore, two vitamin K_2_ homologues, menaquinone-4 (MK-4) and menaquinone-7 (MK-7), have been used as nutrients by the food industry and as nutritional supplements to support bone and cardiovascular health. However, little is known about the bioavailability of nutritional MK-4. To investigate MK-4 and MK-7 bioavailability, nutritional doses were administered to healthy Japanese women.

**Findings:**

Single dose administration of MK-4 (420 μg; 945 nmol) or MK-7 (420 μg; 647 nmol) was given in the morning together with standardized breakfast. MK-7 was well absorbed and reached maximal serum level at 6 h after intake and was detected up to 48 h after intake. MK-4 was not detectable in the serum of all subjects at any time point. Consecutive administration of MK-4 (60 μg; 135 nmol) or MK-7 (60 μg; 92 nmol) for 7 days demonstrated that MK-4 supplementation did not increase serum MK-4 levels. However, consecutive administration of MK-7 increased serum MK-7 levels significantly in all subjects.

**Conclusions:**

We conclude that MK-4 present in food does not contribute to the vitamin K status as measured by serum vitamin K levels. MK-7, however significantly increases serum MK-7 levels and therefore may be of particular importance for extrahepatic tissues.

## Introduction

Vitamin K acts as a cofactor for the endoplasmic enzyme γ- glutamylcarboxylase during the post-translational conversion of glutamic acid residues of specific proteins to γ-carboxyglutamic acid (Gla) to form Gla-containing proteins. A number of blood coagulation factors including coagulation factors II (prothrombin), VII, IX, and X are well-known examples of Gla-containing proteins, which are synthesized in the liver. Osteocalcin, a bone-specific protein synthesized by osteoblasts, and matrix Gla protein synthesized in blood vessel and bone are Gla-containing proteins synthesized at extra-hepatic sites [1].

There are two naturally occurring forms of vitamin K: vitamin K_1_ (phylloquinone) derived from green plants and vitamin K_2_ (menaquinones, MK-n), which is a series of vitamers with multi-isoprene units at position 3 of the common 2-methyl-1,4-naphthoquinone ring structure.

In food, vitamin K_1_ is bound to the chloroplast membrane of leafy green vegetables. MK-4 is found in animal products such as eggs, meat, and liver. MK-4 is derived from the conversion of menadione (synthetic analog of vitamin K only consisting of the 2-methyl-1,4-naphthoquinone ring structure), which is given to the animals. Long chain menaquinones (i.e. MK-7, MK-8, and MK-9) are found in fermented foods such as cheese, curd, and sauerkraut [2]. The Japanese fermented food “natto” contains MK-7 at an exceptionally high concentration [2].

The effects of long chain MK-n such as MK-7 on normal blood coagulation is greater and longer lasting than vitamin K_1_ and MK-4 [3-5]. The effect of natto derived MK-7 was attributed to its very long half-life in serum, providing a better carboxylation-grade of osteocalcin compared to Vitamin K_1_[5].

Recent studies revealed that vitamin K_2_ contributes to both bone and cardiovascular health [6-8]. Both MK-4 and MK-7 have been used as nutritional ingredients. It has been shown that all vitamin K homologues can be converted to MK-4 *in vivo*[9-11]. MK-4 is thought to have specific functions other than γ-carboxylation of vitamin K-dependent proteins [12,13]. However, only little is known about the bioavailability of the nutritional dose of MK-4 [7]. In this study, we compared the bioavailability of MK-4 and MK-7 and subsequent changes in serum levels in healthy volunteers.

## Methods

### Subjects

Ten healthy female volunteers (age: 20–21 years, mean BMI: 20.4 kg/m^2^) not currently taking any medication were selected from the student population of the Laboratory of Physiological Nutrition at Kagawa Nutrition University. Subjects were not allowed to take natto and vitamin supplements other than the experimental versions provided during the study. Approval for both studies was obtained from the Ethics Committee of Kagawa Nutrition University. Informed consent of all volunteers was provided in accordance with the Declaration of Helsinki.

### Study 1

Ten healthy female subjects (age: 20–21 years) were randomized into two groups (n = 5). A single dose of MK-4 (420 μg; 945 nmol) or MK-7 (420 μg; 647 nmol) was administered to each subject within 10 min after ingesting a breakfast containing 13–17 g of fat. All subjects received the same meals, and the nutrients and energy levels were adjusted according to the Japanese Dietary Reference Intake and National Health and Nutrition Examination Survey. The amount of 420 μg is equivalent to the MK-7 concentration in natto and is 7 times higher than the recommended dietary intake (RDI) of vitamin K for Japanese women (age: 19–29 years). Blood (2 ml) was taken to prepare serum before the administration of vitamin K at baseline (t=0), and at 2, 4, 6, 8, 10, 24, 48, and 72 h after administration. Serum MK-4 and MK-7 levels were determined by HPLC analysis as described below.

### Study 2

Ten healthy female subjects (age: 20–21 years) were randomized into two groups (n = 5). MK-4 (60 μg; 135 nmol) or MK-7 (60 μg; 92 nmol), equivalent to the RDI, was administered daily after supper for one week. Subsequently, serum MK-4 and MK-7 levels were determined.

## Materials

MK-4 and MK-7 for the analytical standard were gifted by Eisai (Tokyo, Japan) and Hofmann-La Roche (Basel, Switzerland), respectively. Pure MK-4 (98.5%) and MK-7 (98.7%) used for the human study were prepared by J-Oil Mills (Fukuroi, Japan) with purity determined by HPLC [4]. Preparation consisted of dilution into hydrogenated starch hydrolyze powder, which was packed in gelatin capsules. The capsules were then packed in an aluminum-light-shed bag and kept in a refrigerator until use. After study completion, we re-analyzed and confirmed that neither MK-4 nor MK-7 decreased during storage (data not shown).

### Measurements of serum vitamin K_2_

Serum vitamin K was measured using HPLC with fluorescence detection after on-line, post column zinc reduction, which converts quinone forms of vitamin K into their fluorescent quinol forms, as described previously [5]. Samples were extracted using hexane. Vitamin K1-25 (GLSynthesis Inc., Worcester, MA) was used as internal standard. The detection limit of MK-4 and MK-7 were 16 pg/ml and 40 pg/ml, respectively. Results were expressed as the mean ± standard error (SEM).

## Results

### Study 1

**Figure 1:**
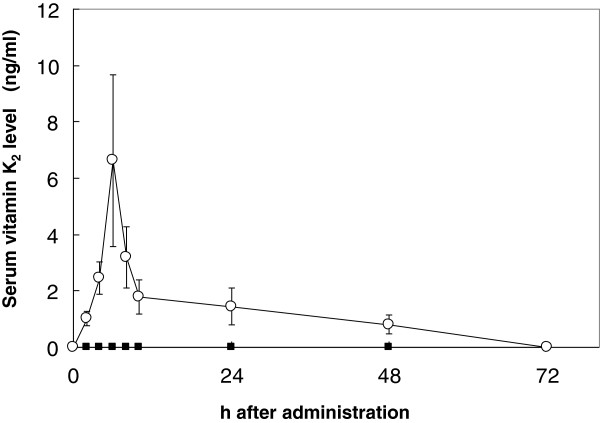
**Change in serum vitamin K**_**2 **_**levels following a single oral dose (420 μg) of MK-4 or MK-7.** Each point represents the mean ± SEM of 5 subjects at 0, 2, 4, 6, 10, 24, 48 and 72 h. ■=MK-4; ○=MK-7

Serum vitamin K_2_ levels were compared after a single oral administration (420 μg) of MK-4 or MK-7 in healthy Japanese females. Baseline serum levels of MK-4 and MK-7 were not detected. Single intake of MK-7 increased serum MK-7 in all subjects, which reached maximum levels at 6 h after administration. MK-7 was detected 48 h after administration (Figure 1). On the contrary, serum MK-4 was not detected at any time point (Figure 1).

### Study 2

**Figure 2:**
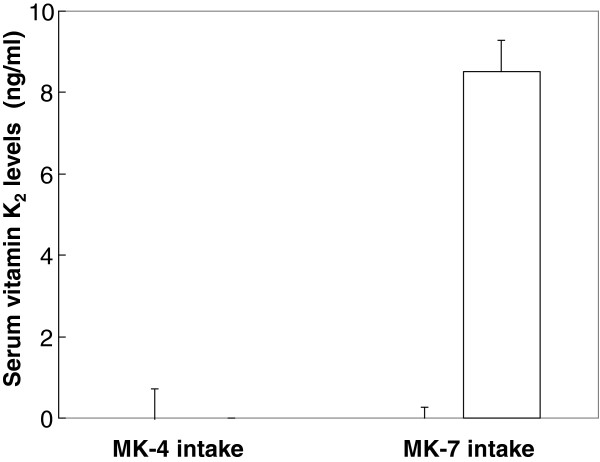
**Increased serum vitamin K**_**2 **_**levels in subjects after 7 days of consecutive administration (60 μg/day).** Each value is expressed as the mean ± SEM of 5 subjects. ■=MK-4;□=MK-7

Serum vitamin K_2_ levels were compared after consecutive administration of MK-4 and MK-7 (60 μg/day) for 7 days. Baseline serum level of MK-4 was 2.2 ng/ml ± 0.38 and that of MK-7 was less than detection limit. After subtracting the baseline serum levels from all values, MK-4 levels were 0.00 ng/ml ± 0.77 and 0.03 ng/ml ± 0.27 in the MK-4 and MK-7-treated groups, respectively. While MK-4 intake did not increase MK-4 administered group, serum MK-7 increased significantly in MK-7-administered-subjects (Figure 2).

## Discussion

The current study shows that MK-4 has a poor bioavailability at a nutritional level dose, whereas MK-7 is well absorbed and detectable in the blood at nutritional levels. In a study from the Netherlands, they compared the absorption of 900 μg of vitamin K_1_, MK-4, and MK-9. MK-4 showed a short serum half-life and small area under the curve compared to vitamin K_1_, whereas MK-9 displayed a long serum half-life compared to vitamin K_1_ or MK-4 [14]. Takeuchi *et al.*[15] reported a dose finding study of MK-4 to increase osteocalcin carboxylation in healthy subjects. In their study, supplementation of 500 μg MK-4/day for 2 months showed no effects on carboxylation of osteocalcin, whereas a dose of 1500 μg MK-4/day was required to improve carboxylation of osteocalcin. From these and our data we can conclude that MK-4 intake of greater than 420–500 μg is required.

Consecutive MK-4 supplementation did not increase plasma MK-4 levels whereas MK-7 supplementation significantly increased plasma MK-7 levels in healthy female subjects. This is in-line with previous published works, which indicated nutritional doses of MK-7 (45–90 μg/day) to be effective for carboxylation of osteocalcin [16,17].

Because all vitamin K homologues can be converted to MK-4 *in vivo*, MK-4 is considered to have specific functions other than γ-carboxylation of vitamin K-dependent proteins [9-11]. However, in a previous rat study from our group [18], the intake of a nutritional dose of MK-4 did not increase the MK-4 levels in extrahepatic tissues, whereas MK-7 significantly increased MK-4 in extrahepatic tissues. Thus, MK-7 is a better supplier for MK-4 *in vivo* than MK-4 itself.

In this study, we demonstrated that a nutritional dose of MK-7 is well absorbed in human, and significantly increases serum MK-7 levels, whereas MK-4 had no effect on serum MK-4 levels. Therefore, the nutritional values of vitamin K_2_ homologues should be differentiated with regard to bioavailability and efficacy.

## Abbreviations

MK-4: Menaquinone-4; MK-7: Menaquinone-7; RDI: Recommended dietary intake.

## Competing interests

TS works for J-OIL MILLS, INC. Other authors have no competing interest.

## Authors’ contributions

All authors contributed in the study design. TS and LS were responsible for data collection and analysis. KU was responsible for management of the human clinical study. All authors read and approved of the final manuscript.
